# Fundamental pharmacological expressions on ocular exposure to capsaicin, the principal constituent in pepper sprays

**DOI:** 10.1038/s41598-018-30542-2

**Published:** 2018-08-14

**Authors:** Harshita Krishnatreyya, Hemanga Hazarika, Achintya Saha, Pronobesh Chattopadhyay

**Affiliations:** 1 Division of Pharmaceutical Technology, Defence Research Laboratory (DRL), Defence Research and Development Organization (DRDO), Tezpur, Assam India; 2 Pharmaceutical and Fine Chemical division, Department of Chemical Technology, University of Calcutta, Kolkata, India

## Abstract

Eye irritation assessment is compulsory to anticipate health risks in military personnel exposed to riot control agents such as capsaicin, the principal constituent of oleoresin capsicum, or pepper sprays. The present work investigates certain fundamental yet unaddressed pharmacological manifestations on ocular exposure to capsaicin. Ocular pharmacology of capsaicin was studied using acute eye irritation (AEI), bovine corneal opacity and permeability (BCOP) assay, corneal fluorescein staining and indirect ophthalmoscopy studies, transcorneal permeation, Schirmer tear secretion test, nerve conduction velocity study and enzyme-linked immunosorbent assay (ELISA). Additionally, histopathology and scanning electron microscopy (SEM) of bovine corneas and rat optic nerves were done to further estimate capsaicin induced morphological variations. Our findings demonstrated that AEI, BCOP, corneal fluorescein staining and indirect ophthalmoscopy were useful in assessing capsaicin induced ocular irritation; AEI and BCOP also contributed towards indicating the eye irritation potential of capsaicin as per the United Nations Globally Harmonized System of Classification and Labelling of Chemicals categorization. Additional experimental observations include considerable transcorneal permeation of capsaicin, capsaicin induced reduction in tear secretions and nerve conduction velocity and increased expression of proinflammatory cytokines by ELISA. Histopathology and SEM were favourable techniques for the detection of capsaicin induced ocular physiological modifications.

## Introduction

Oleoresin capsicum (OC) incorporated military defense sprays were first developed as an alternative to chloroacetophenone and *o*-chlorobenzylidene malononitrile sprays in the 1970s^1^. OC or pepper sprays are used by government defence organizations worldwide as non-lethal incapacitating agents for law enforcement, against interpersonal violence or civil unrest, criminal incapacitation etc. Capsaicin, a naturally occuring compound, is the principal pungent constituent amongst other vanillylamides present in OC^2,3^. It is a known sensory irritant and some typical ocular manifestations on exposure to capsaicin are pain, burning sensation, tearing and blepharospasm, conjunctivitis etc depending on the dose and duration of exposure^4–6^.

The evident utility of capsaicin as a riot control agent for military defence organizations is irrefutable^7^ and the assessment of exposure risks associated with it, is therefore, essential. Capsaicin is classified as category 2 A, i.e, it induces reversible eye irritation, based on the United Nations Globally Harmonized System of Classification and Labelling of Chemicals (UN GHS) categorization in accordance with Occupational Safety and Health Administration – Hazard Communication Standard 29 CFR 1910^8^. The UN GHS construes eye irritation as alterations in the eye after administration of a test substance to its outer surface, which is completely reversible within 21 days^9^. Recommendation of a sequential experimental strategy for examination of acute eye irritation/corrosion induced by different chemicals was first done by the Organization for Economic Cooperation and Development (OECD) who adopted the Guidelines for the Testing of Chemicals causing acute eye irritation/corrosion (TG 405) in 1981^10^. Data on the overall toxicology of OC is extant particularly regarding effects following exposure to OC via the inhalation route^1^. Capsaicin, despite being a known ocular irritant, has generated little data on mammalian eye corrosion/irritation and hence, was tested mainly with the aim that the testing strategy might generate information about specific pharmacological manifestations in addition to confirming its UN GHS categorization.

The Bovine Corneal Opacity and Permeability assay, (BCOP), was adopted by the OECD in 2009, to identify ocular corrosives and serious irritants as mentioned in the OECD Test Guideline 437^11^. The BCOP test is an organotypic archetype and assures short-term sustenance of the common physiological as well as biochemical environment of the bovine cornea *in vitro*^12^. Corneal deterioration is determined through quantitative assessment of modifications in corneal opacity and permeability with an opacitometer and a visible light spectrophotometer subsequently^13^. The BCOP assay was adopted to determine possibilities of capsaicin induced opacity/transparency and permeability on bovine corneal models and to confirm its adherence to the UN GHS categorization.

In addition to the acute eye irritation (AEI) and BCOP assay, specialized pharmacological testing procedures were utilized for the assessment of capsaicin induced ocular irritation. Corneal fluorescein staining and indirect opthalmoscopy were done to check for the validation of irritation symptoms within the eye. Tears are a natural phenomena providing lubrication and protection to the ocular surface^14^. Capsaicin induced alterations in tear secretions were measured by the Schirmer tear secretion test. Transcorneal permeation of the lipophilic capsaicin sample across bovine corneal membrane was studied to quantify capsaicin passage and factors that influence it. Electrophysiological study comprising of the nerve conduction velocity (NCV) study of rat optic nerves was done to assess variations in the conduction of nerve impulses across the optic nerve, which is accountable for the transmission of visual information to the brain^15^. Histopathology and scanning electron microscopy (SEM) of bovine corneas and optic nerves were done to evaluate, in further detail, any morphological variations induced by capsaicin. Fluctuations in the proinflamamtory cytokines IL-1α, IL-1β, IL-6 and TNF-α due to capsaicin exposure was measured by the enzyme-linked immunosorbant assay (ELISA).

## Results and Discussion

### Acute eye irritation

**Figure 1:**
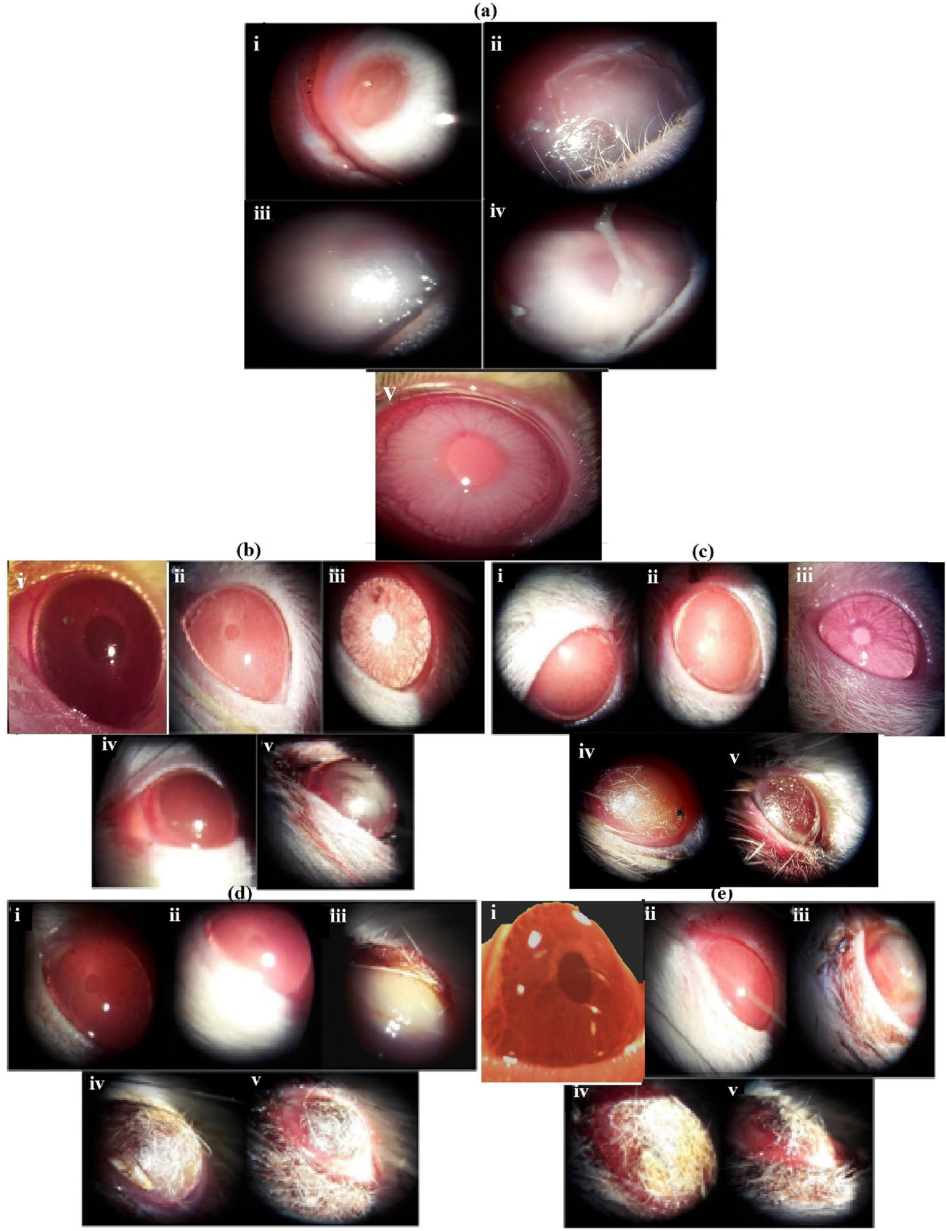
Acute eye irritation (**a**) Rabbit eyes (i) normal and 50 mg/ml capsaicin treated eyes in (ii) 1 hr (iii) 24 hrs (iv) 48 hrs and (v) 72 hrs. Severity of ocular lesions have been scored in Table 1. (**b**) Rat eyes (i) normal and capsaicin treated (ii) 25 µg/ml, (iii) 50 µg/ml, (iv) 75 µg/ml and (v) 100 µg/ml at 1 hr after exposure. For all the rat eye irritation observations, severity of ocular lesions have been scored in Table 2. (**c**) Rat eyes (i) normal and capsaicin treated (ii) 25 µg/ml, (iii) 50 µg/ml, (iv) 75 µg/ml and (v) 100 µg/ml at 24 hrs after exposure. (**d**) Rat eyes (i) normal and capsaicin treated (ii) 25 µg/ml, (iii) 50 µg/ml, (iv) 75 µg/ml and (v) 100 µg/ml at 48 hrs after exposure. (**e**): Rat eyes (i) normal and capsaicin treated (ii) 25 µg/ml, (iii) 50 µg/ml, (iv) 75 µg/ml and (v) 100 µg/ml at 72 hrs after exposure.

Ocular irritation in rabbits exposed to 50 mg/ml capsaicin was determined by scoring/grading the ocular lesions produced in the animals (in accordance with the TG 405 guidelines) as shown in Table 1. A slit lamp microscope (Haagstreit type AIA-11, Appaswamy) was used in grading as well as evaluating the ocular endpoints. Figure 1(a) exhibits the immediate characteristic observations in rabbits including erythema of the eye, tear secretions, corneal and conjunctival abrasion, swelling, opacification and thickening. Observations were made after 1, 24, 48 and 72 hrs. The irritation effects subsided with respect to increasing time. In rats, treatments of 25, 50, 75 and 100 µg/ml capsaicin instilled in the eyes caused evident pain, convulsive blinking and produced lesions which were graded and scored for a time period of 1–72 hrs as shown in Table 2. Aberrant permeability of the eyelids and conjunctival blood vessels was observed in rats. Severity of lesions was conceivably higher in animals exposed to higher doses of capsaicin and improved with increasing time [Fig. 1(b–e)]. Subsidation of irritation symptoms was observed in rats treated with 25 µg/ml capsaicin. However, rats exposed to 50, 75 and 100 µg/ml capsaicin showed symptoms of irreversible ocular pain and distress and were euthanized without much delay.

**Table 1:** Acute eye irritation (AEI) in rabbits: Grading of ocular lesions was done as per OECD 405 grading guidelines. Figures indicate the individual gradings of the 6 animals used in the experiment.

Sl no	Animal species	Capsaicin exposure	GRADING OF OCULAR LESIONS															
			Response after															
			1 hr				24 hrs				48 hrs				72 hrs			
			Cornea	Iris	Conjunctiva	Chemosis	Cornea	Iris	Conjunctiva	Chemosis	Cornea	Iris	Conjunctiva	Chemosis	Cornea	Iris	Conjunctiva	Chemosis
1	Rabbit (*Oryctolagus cuniculus*)	50 mg/ml	3	0	2	2	2	0	1	0	1	0	1	0	1	0	1	0
2			3	2	2	2	2	1	0	1	1	0	1	0	1	0	1	0
3			3	2	2	1	2	0	0	1	1	1	1	0	1	1	1	0
4			3	1	2	2	2	0	1	1	1	0	1	0	1	0	0	0
5			3	2	1	1	2	0	0	0	1	0	0	0	1	1	1	0
6			3	1	2	2	2	1	1	1	1	1	1	0	1	1	1	0

**Table 2:** AEIin rats: Grading of ocular lesions was done as per OECD 405 grading guidelines.

Sl no	Animal species	Capsaicin treatment received	GRADING OF OCULAR LESIONS															
			Response after															
			1 hr				24 hrs				48 hrs				72 hrs			
			Cornea	Iris	Conjunctiva	Chemosis	Cornea	Iris	Conjunctiva	Chemosis	Cornea	Iris	Conjunctiva	Chemosis	Cornea	Iris	Conjunctiva	Chemosis
1	Wistar rats (*Rattus norvegicus*)	Control	0	0	0	0	0	0	0	0	0	0	0	0	0	0	0	0
2		25 µg	2 ± 0.6	1.1 ± 0.4	1.1 ± 0.4	1.1 ± 0.4	0	0	0	0	0	0	0	0	0	0	0	0
3		50 µg	2.6 ± 0.8	1.3 ± 0.5	2.3 ± 0.5	2.1 ± 0.4	1.3 ± 0.5	0	1.1 ± 0.4	0	3.1 ± 0.4	0	2.1 ± 0.6	0	3.8 ± 0.4	0	3.8 ± 0.4	2.1 ± 0.4
4		75 µg	2.6 ± 0.5	1.5 ± 0.5	1.6 ± 0.5	1.8 ± 0.4	2.6 ± 0.5	0	2.1 ± 0.4	1.1 ± 0.2	3.8 ± 0.4	0	2.1 ± 0.4	1 ± 0.0	4 ± 0.0	0	3.6 ± 0.5	2 ± 0.0
5		100 µg	3.5 ± 0.5	2.1 ± 0.4	2.6 ± 0.5	3.1 ± 0.4	3.8 ± 0.4	1.8 ± 0.4	3.1 ± 0.4	2 ± 0.0	4 ± 0.0	0	2.6 ± 0.5	1 ± 0.0	3.8 ± 0.4	0	3.5 ± 0.5	2.1 ± 0.6

Numbers indicate mean grading of the 6 animals used per group in the experiment, i.e, mean ± S.D.

Very little experimental data reporting detailed capsaicin induced acute eye irritation has been discussed and/or studied previously. In this regard, there has been a constant focus to develop alternate testing strategies to the regular Draize toxicity tests^16^. The progress of such alternative models have, however, not advanced in a substantial manner as of now^17^. A test substance is categorized as UN GHS eye irritant Category 2A (irritating to eyes) when it shows in at least 2 of 3 experimental animals, a positive response of: (i) corneal opacity ≥1; and/or (ii) iritis ≥1; and/or (iii) conjunctival redness ≥2; and/or (iv) conjunctival edema (chemosis) ≥2 estimated as the mean scores after grading at 24, 48 and 72 hours following instillation of the test substance, and which entirely reverses within an observation time of 21 days. On the other hand, an eye irritant is categorized as Eye Category 2B (irritating to eyes) when the effects mentioned above are entirely reversible within an observation time of 7 days^8^. In both rabbits and rats, corneal swelling and erythema were distinctly reduced within the 72 hours and were evidently reversible. Rabbits exposed to 50 mg/ml and rats treated with 25 µg/ml capsaicin showed reversibility of capsaicin induced responses before 7 days of exposure to capsaicin. Thus, on experimental basis, our results categorize capsaicin as an irritant belonging to the Category 2B instead of Category 2A, as the reversibility of animal responses (both rabbits and rats) were observed within 7 days, instead of 21 days.

### Histopathology

**Figure 2:**
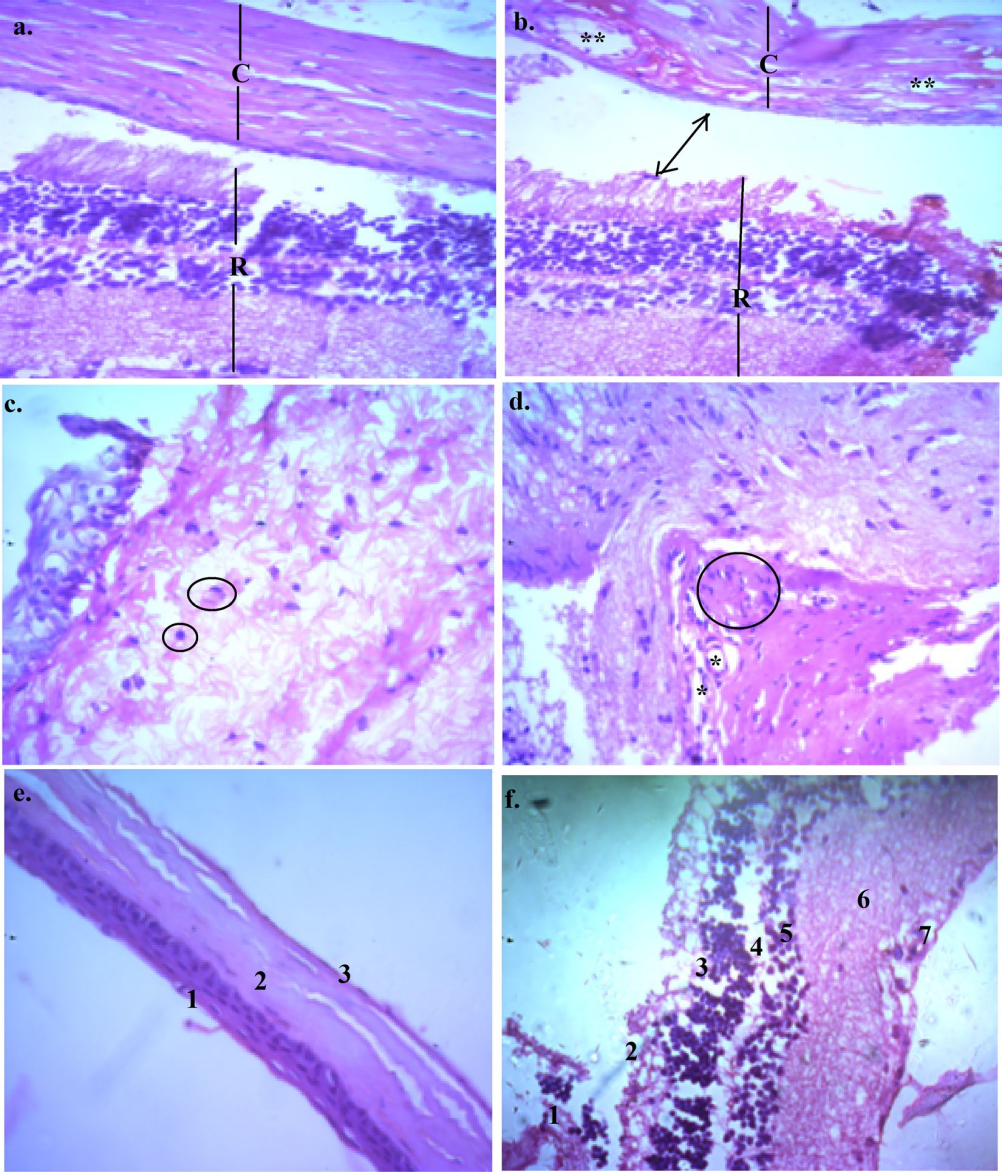
Haematoxylin & eosin (H & E) stained rat eye histopathology using light microscope at 40X magnification (**a**) cross section of a normal rat corneal stroma (C) and retina (R) (**b**) 25 µg/ml capsaicin treated corneal and retinal section showed possible corneal separation and disfiguration from the retina (marked with an arrow) along with corneal vacuolation and disfiguration (marked as^**^) (**c**) control corneal stroma with circled areas depicting nuclei of the stromal cells (**d**) 50 µg/ml capsaicin treated corneal stroma depicting stromal swelling, nuclear aggregation (circled) and vacuolar seperations (indicated by asterisks) (**e**) 100 µg/ml capsaicin treated corneal section with distinct inflammation in the (1) stratified squamous epithelia containing wing and basal cells, (2) corneal stroma with occasional cracks in it, (3) very indistinct posterior limiting membrane (**f**) 100 µg/ml capsaicin H & E treated retinal section magnification showing capsaicin induced variations in (1) choroid, (2) retinal pigment epithelium, (3) outer nuclear layer, (4) outer plexiform layer, (5) inner nuclear layer, (6) inner plexiform layer, (7) retinal ganglion cell layer. Observations here include depletion of the ganglion cell layer (7) while the plexiforn layers (4 and 6) remained indistinct. Very little broken choroidal epithelia (1) could be seen which did not form a continous layer.

Histopathological examinations was done using a light microscope (Axio Scope A1 Carl Zeiss, Germany) and comparisons of control and 25, 50, 75 and 100 µg/ml of capsaicin treated rat eyes showed marked disorganization of the corneal epithelia and stroma. All histopathological observations have been made at 40X magnification. Figure 2a shows the corneal stroma (marked as “C”) and what appears to be a part of the retina (marked as “R”) with quite an orderly lamellar architecture. Eyeball sections of 25 µg/ml capsaicin treated cornea and retina (Fig. 2b) shows corneal stromal disfiguration and vacuolar gaps within the tissues which might be an indication of stromal edema (due to inflammatory reaction) and increased stromal fluid thereby increasing corneal hydration and opacity. Though a bit disfigured, the retina did not seem much affected and maintained more or less intact layers. Figure 2c shows a portion of the normal corneal stromal cells with nuclei (circled areas) whereas Fig. 2d shows the stromal section treated with 50 µg/ml capsaicin thereby resulting in prominent vacuolar separations (asterisk marked), nuclear aggregation, inflammation and stromal swelling. Clefts/vacuoles in the corneal stroma (marked as “2” in the image), very thin and almost invisible Descemet’s membrane (marked as “3” in the image) were observed in 100 µg/ml capsaicin treated cornea, Fig. 2e. Figure 2f shows 100 µg/ml capsaicin treated retinal tissues with depletion of the ganglion cell layer (marked as “7” in the image) and the plexiforn layers (marked as “4” and “6”) remaining indistinct. Very little broken choroidal epithelia (marked as “1” in the image) could be seen which did not form a continuos layer.

Histopathology results demonstrated increased and prominent rat corneal disfigurations with increased doses of capsaicin without significant effects in the retinal tissue at lower doses, a possible explanation for which might be the capsaicin exposure method and duration of exposure. Our reports on capsaicin have suggested disorganization and separation of the stromal collagen lamellae, stromal neovascularisation and Descemet’s membrane folding due to edematous cornea^18^, some of which were evident from our results. The alterations detected in our study were, to some extent, similar to symptoms such as epithelial alteration, stromal edema and scar formation observed in capsaicin induced neurotrophic keratouveitis in young rats^19,20^.

### Bovine corneal opacity and permeability (BCOP) assay

**Figure 3:**
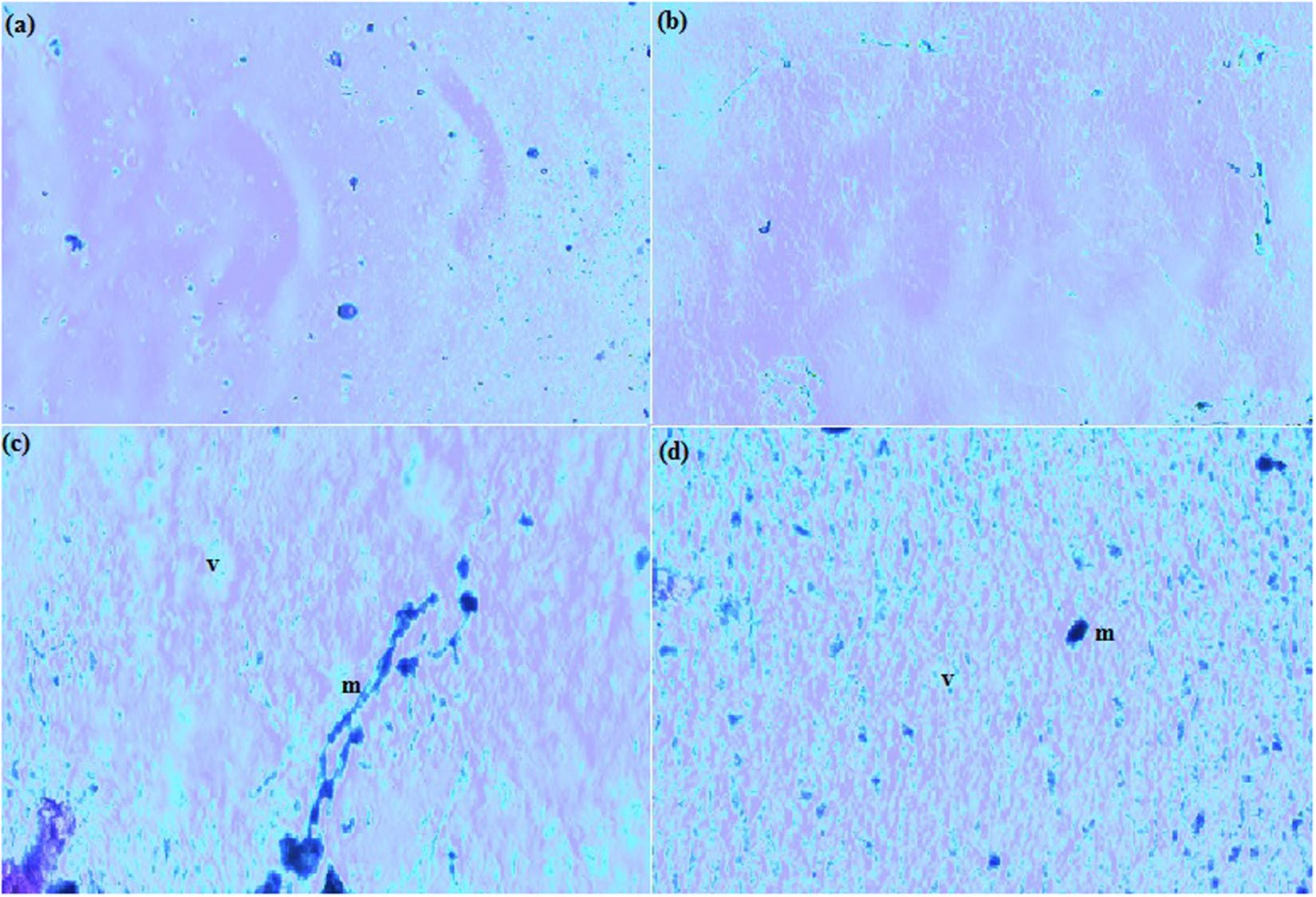
Bovine corneal opacity and permeability assay (**a**) Light microscope observations of H & E stained histopathology sections at 40X of bovine corneas exposed to (**a**) PBS 7.4 and capsaicin treated (**b**) 5 mg/ml, (**c**) 25 mg/ml and (**d**) 50 mg/ml. As suggested Images suggest certain features such as cytoplasmic breakdown, vacuolar gaps (marked as “v”) and corneal mineralization (marked as “m”) were more prominent in corneas exposed to higher doses of capsaicin. Slight cytoplasmic breakdown can be also observed in 3(b) which is more conspicuous in Fig. 3(c,d), thereby indicating symptoms of predominant inflammation.

The eye-irritation potential of 5, 25 and 50 mg/ml of capsaicin was measured by its ability in generating increased opacity and reduced permeability in isolated bovine corneas as indicated in Table 3. Prominent variations in the opacity and permeability of the corneal membranes on exposure to the different concentrations of capsaicin was evidently predictable. Corneal opacity elevated with increasing capsaicin concentrations, which is obvious from the opacity values observed in Table 3. Textural changes such as wrinkling and dehydration of the corneal membranes exposed to capsaicin are apparent and might influence its permeability thereby either mediating or inhibiting further passage of materials into the anterior segment of the eye. Fluorescein retention/leakage across the corneal membrane was minimal. No significant permeability of sodium fluorescein (4 mg/ml) was observed after corneal exposure to capsaicin treatments. The volume of sodium fluorescein dye that permeated through corneal membrane after exposure to capsaicin was totally insignificant and generated absorbance values of 0.024, 0.010 and 0.008 for capsaicin treatments of 5, 25 and 50 mg/ml when compared to negative and positive controls which showed values of 0 and 1.120 respectively. We can hereby conclude that higher doses of capsaicin increases opacity while decreasing the permeability of the corneal membrane. A light microscopic evaluation of the 5, 25 and 50 mg/ml of capsaicin treated bovine corneas showed damage and loss of superficial epithelial lining. Mild cytoplasmic vacuolations (marked as “v”) in both capsaicin treated corneas and to a little extent, in Phospahte buffer solution 7.4 (PBS) buffer treated corneas were recorded in Fig. 3(a–d) which seemed to increase with increasing irritant doses [Fig. 3(b–d)]. Slight breakdown of stromal cytoplasm in capsaicin treated corneas were observed. There is a possibility of capsaicin induced corneal mineralization (marked as “m”) in treated bovine corneas as irregular aggregates (bluish colorations) were present within the stromal cytoplasm.

**Table 3:** Bovine corneal opacity and permeability. Opacity and permeability values of bovine corneas as per the OECD BCOP 437 guidelines.

Sl no	Exposure	Opacity	Permeability	IVIS	UN GHS
1	Negative control^*^	3 ± 0.28	0.00	3 ± 0.28	No category
2	5 mg/ml	18.28 ± 0.12	0.024 ± 0.22	18.64 ± 0.19	No prediction can be made
3	25 mg/ml	19.01 ± 0.09	0.010 ± 0.41	19.37 ± 0.25	No prediction can be made
4	50 mg/ml	19.85 ± 0.31	0.008 ± 0.19	19.97 ± 0.46	No prediction can be made
5	Positive control^*^	50.22 ± 0.16	1.120 ± 0.34	67.02 ± 0.45	Category 1

^*^Negative control = Phosphate Buffer 7.4.

^*^Positive control = Benzalkonium chloride, 5%.

Little preceding surveys reporting the corneal opacity and permeation induced by capsaicin have been conducted  earlier. According to a study by Ogilvy *et al*.^21^, capsaicin treated rats showed corneal lesions which differed from numerous punctuate extents of corneal opacity to deep stromal opacity with ulceration as well as neovascularisation^21^. Our BCOP results distinctly confirm corneal opacification on exposure to varied doses of capsaicin thereby influencing the transparency of the corneal membrane and affecting its permeability either facilitating or reducing the passage of materials across it. According to Swan *et al*.^22^, they observed the higher corneal penetration of non polar compounds like oils and fats, as compared to hydrophobic and polar compounds^22^. Capsaicin, being a non polar lipophilic compound, is thus expected to show considerable penetration across the corneal membrane. The results of our BCOP study confirm increased opacity and decreased corneal permeability with the passage of increased doses of capsaicin. An *In Vitro* Irritancy Score (IVIS) was useful in allocating the irritancy level of a test group and was calculated as given below:

$${\rm{IVIS}}={\rm{mean}}\,{\rm{opacity}}\,{\rm{value}}+({\rm{15}}\times {\rm{mean}}\,{\rm{permeability}}\,{\rm{value}})$$

Classification of eye irritation by the BCOP is as follows: a test substance that indicates an IVIS ≤ 3 is classified as UN GHS No Category; indication of 3 < IVIS ≤ 55 is classified as UN GHS No prediction can be made; and an indication of IVIS > 55 is classified as UN GHS Category 1 (corrosive or severe irritant)^12^. Our observations on 5, 25 and 50 mg/ml treatments of capsaicin brought us to conclusion that “no prediction could be made” (Table 3). BCOP assay seems to identify capsaicin as Category 2 as it has a mean IVIS that is nearer to 3 but lesser than 55 and produces pathological damages to the eye and its categorization remains similar to that of the UN GHS.

Cooper *et al*.^23^ have earlier determined the extent of damage that a substance could produce in the cornea by using histopathology as a supplementary endpoint. Histopathology provides important information about the forms of lesions, in addition to using opacity or permeability study^23^. As studied by Meador *et al*.^24^, corneal mineralization might occur due to trauma or inflammation, or as a result from spontaneous exposure to topical irritants^24^ (capsaicin, in this case). Our observations are in consideration with Gallar *et al*.^25^, who observed corneal mineralization characterized by corneal dystrophy or calcific keratopathy that occured spontaneously in rats and mice exposed to capsaicin^25^.

### Corneal fluorescein staining

**Figure 4:**
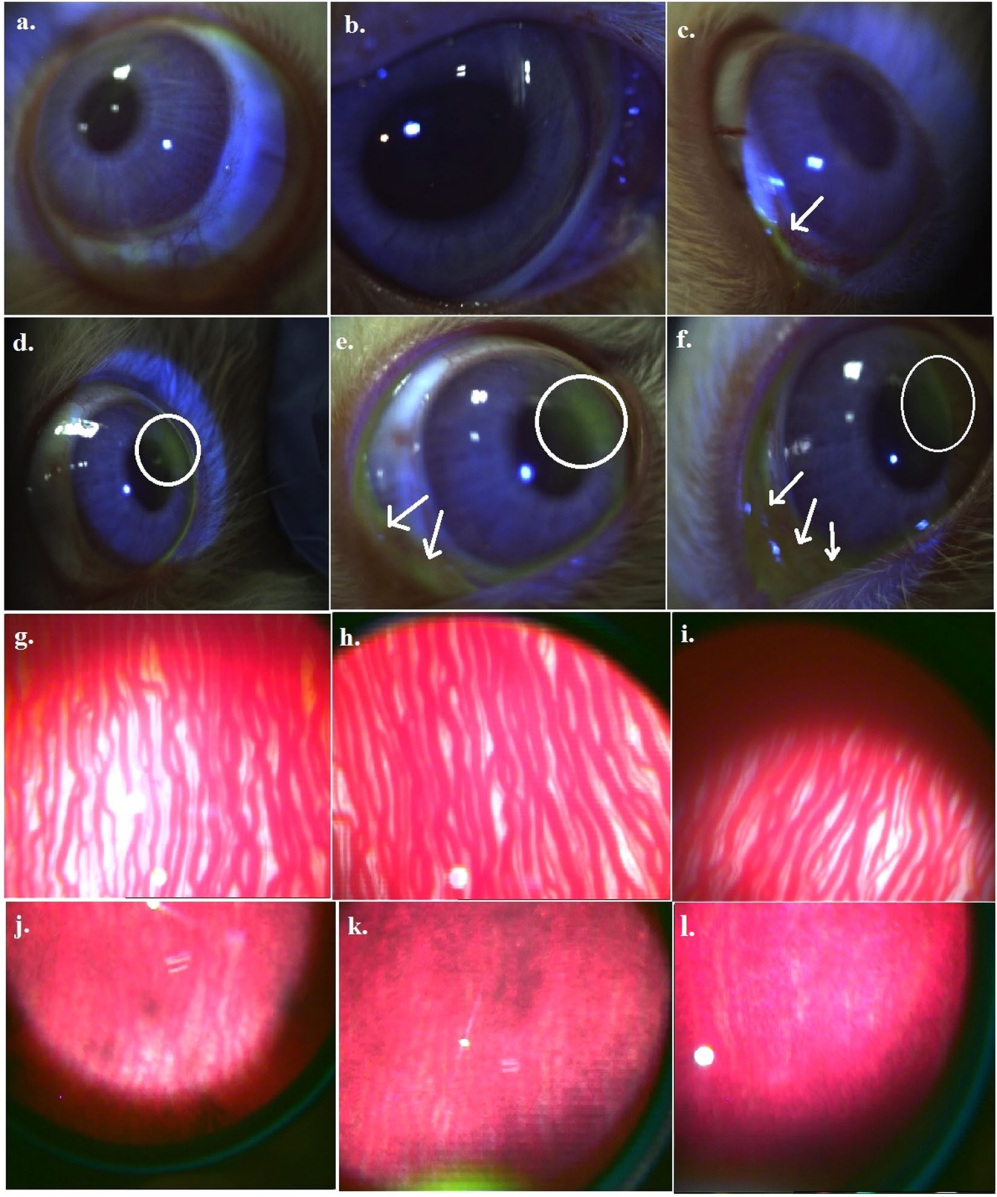
Corneal fluorescein staining in rabbit eyes (**a**) control (**b**) DMSO exposed (**c**) 25 mg/ml capsaicin exposed (**d**) 50 mg/ml capsaicin exposed (**e**) 75 mg/ml capsaicin exposed (**f**) 100 mg/ml capsaicin exposed. There was no fluorescein uptake by the control and vehicle treated cornea (4a and 4b) whereas minute staining was observed in the conjunctiva of rabbit exposed to 25 mg/ml capsaicin (Fig. 4c marked with an arrow). Eyes of animals exposed to 50, 75 and 100 mg/ml capsaicin (Fig. 4d–f) showed fluorescein staining in minute areas of the iris (marked in circles) and conjunctiva (marked using arrows) followed by diminished symptoms later. Indirect opthalmoscope imaging of rabbit fundus using a 20D lens: (**g**) normal rabbit eye with retinal blood vessels in vitreous media (**h**) DMSO exposed fundus (**i**) 25 mg/ml capsaicin exposed fundus (**j**) 50 mg/ml capsaicin exposed fundus (**k**) 75 mg/ml capsaicin exposed fundus (**l**) 100 mg/ml capsaicin exposed fundus. Control, DMSO and 25 mg/ml capsaicin treated fundus (Fig. 4g–i) comprising of retinal blood vessels and vitreous media remained clear and distinct whereas 50, 75 and 100 mg/ml capsaicin treated eyes (Fig. 4j–l) showed vitreous haze with only slight visibility of the blood vessels which might be as a result of vascular inflammatory granulation (observed as dark patches in images Fig. 4j–l) and diffuse retinal oedema observed to a greater extent in fundus exposed to higher doses of capsaicin.

Fluorescein eye staining testing is a beneficial and favourable application in appraising corneal epithelial irregularities of patients since it provides essential information concerning the degree and/or extent of tissue injury^26,27^. Scoring of fluorescein stained corneas exposed to different treatments have been shown in Table 4. Figure 4(a–f) show images of corneal fluorescein staining on control, vehicle treated (i.e, DMSO) and 25, 50, 75 and 100 mg/ml of capsaicin on rabbit eyes. Both the control and DMSO treated cornea exhibited no fluorescein uptake (Fig. 4a,b) whereas fluorescein uptake by capsaicin treated inflammed corneas was detected immediately after exposure which indicated the existence of mild epithelial damage and abnormalities within the iris and conjunctival areas (Fig. 4c,f). Corneas exposed to 25 and 50 mg/ml capsaicin (Fig. 4c,d) showed reduced corneal uptake of fluorescein with very slight staining in minute areas of the conjunctiva and iris. Corneas exposed to 75 and 100 mg/ml capsaicin (Fig. 4e,f) produced staining in the iris and conjunctiva as well, but predictably to a greater extent as compared to the lower doses of capsaicin. Thus results of corneal fluorescein staining test depicted capsaicin induced nominal corneal damage in rabbits which diminished with respect to time. Since the test revealed no profound damage by ocular capsaicin, further scoring after 24 hrs was discontinued.

**Table 4:** Grading of corneal fluorescein staining in rabbit eyes.

Sl no.	Treatments		GRADING OF CORNEAL FLUORESCEIN STAINING (RABBITS)					
			Response after					
			1 hr			24 hrs*		
			Cornea	Nasal conjunctiva	Temporal comnjunctiva	Cornea	Nasal conjunctiva	Temporal comnjunctiva
1	Control		0	0	0	0	0	0
			0	0	0	0	0	0
			0	0	0	0	0	0
2	DMSO		0	0	0	0	0	0
			0	0	0	0	0	0
			0	0	0	0	0	0
3	Capsaicin	25 mg/ml	0	1	0	0	0	0
			0	1	0	0	0	0
			0	1	0	0	0	0
4		50 mg/ml	1	0	0	0	0	0
			1	0	0	0	0	0
			1	0	0	0	0	0
5		75 mg/ml	1	1	0	0	0	0
			1	1	0	0	0	0
			1	1	0	0	0	0
6		100 mg/ml	1	2	0	0	1	0
			1	2	0	0	1	0
			1	2	0	0	1	0

^*^No significant fluorescein uptake was observed at 24 hrs and hence, grading of images at 24 hrs are based on experimental observations that are not significant and have not been shown.

### Indirect ophthalmoscope imaging

For the evaluation of capsaicin induced morphological variations in the fundus, vitreous and blood vessels of the posterior eye, control, DMSO exposed, 25, 50, 75 and 100 mg/ml capsaicin exposed rabbit eyes were observed using a Keeler Vantage Indirect Ophthalmoscope (Keeler Ophthalmic Instruments, USA). Indirect opthalmoscope imaging is a favoured technique for comprehensive eye examination, as it provides the examiner with an improved and extensive view of the inner eye^28,29^. The fundus comprising of retinal blood vessels and vitreous media remained clear, distinct and there were no retinal detachments in the control, DMSO and 25 mg/ml capsaicin exposed eyes as observed in Fig. 4g–i. Contrary to that, 50, 75 and 100 mg/ml capsaicin treated eyes (Fig. 4j–l) showed indistinct fundus images with only slight visibility of the blood vessels accompanied by vitreous haze. This might be due to vascular inflammatory granulation (observed as dark patches in images Fig. 4j–l and diffuse retinal oedema observed to a greater extent in fundus exposed to higher doses of capsaicin as compared to the control, vehicle treated and 25 mg/ml capsaicin exposed fundus. Thus, indirect opthalmoscope imaging demonstrated prominent vascular changes in the ocular tissues and it can be concluded that when administered topically, capsaicin (considering the dosage and duration of exposure) is likely to elicit substantial damaging response in the inner eye.

### Schirmer tear secretion test

**Figure 5:**
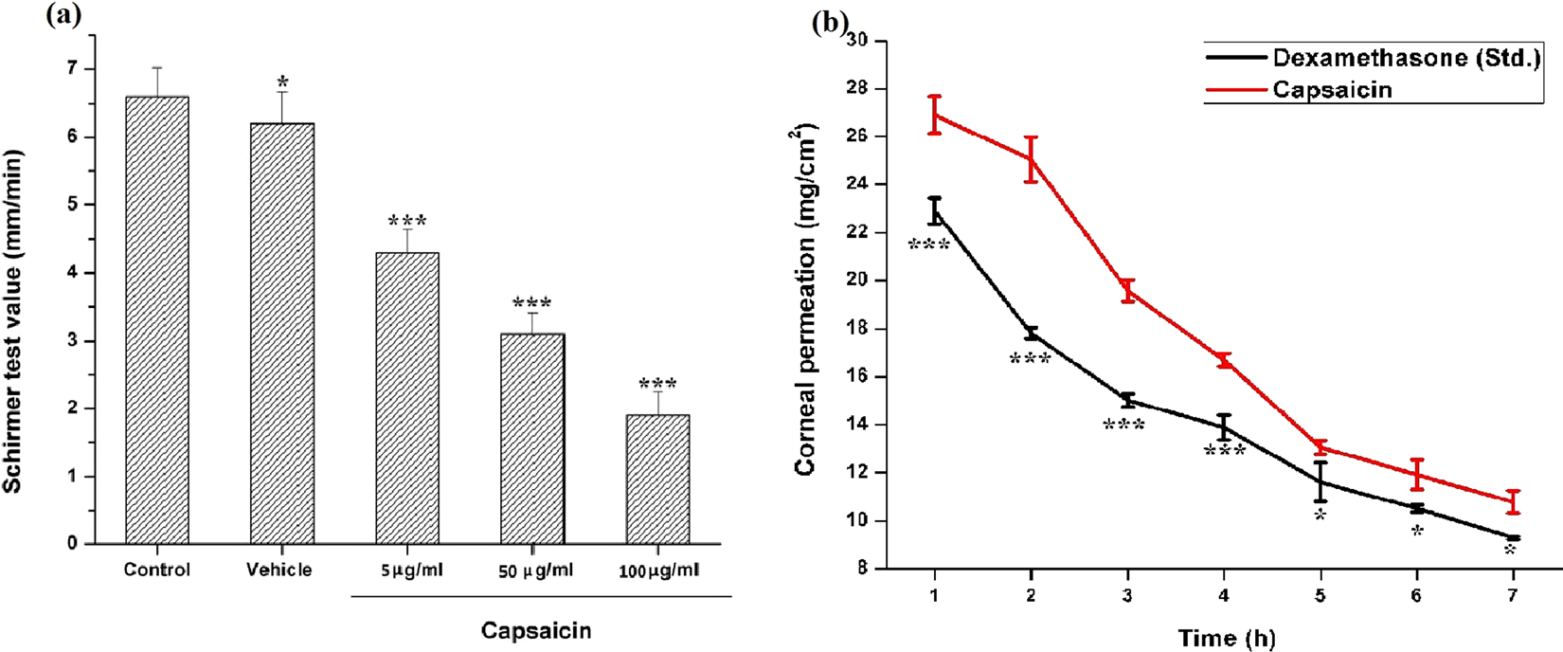
(**a**) Schirmer tear secretion test results: Graphs indicate variations in tear secretion induced by 5, 50 and 100 µg/ml of capsaicin exposure in rats. Each column and vertical bar shows the mean ± standard deviation (n = 6). An asterisk indicates statistically significant differences (^***^P < 0.001, ^*^P < 0.05). Statistically significant differences were observed in capsaicin treated groups when compared with the control groups. Transcorneal permeation profile of 50 mg/ml capsaicin versus 50 mg/ml of dexamethasone (standard): (**b**) Capsaicin and dexamethasone showed similar and rapid permeation across the corneal membranes initially until the 1^st^ hour, which was followed by a linear and gradual decrease later. The permeability rate exhibited rapid and fast absortion of about 53.8% capsaicin (26.9 mg/cm^2^) as compared to 45.8% (22.9 mg/cm^2^) dexamethasone in the first hour. Earlier studies suggest considerable permeation and absorption of dexamethasone when formulated in cyclodextrine microparticle carrier systems via the ocular topical route^31^. Values are mean ± standard deviation (*n* = 4). Statistically significant differences were observed in capsaicin treated groups when compared with the control group (^***^P < 0.001, ^*^P < 0.05).

As shown in Fig. 5(a), capsaicin provoked a dose dependent reduction of tear fluid secretion in rats. Significant reductions (P < 0.05) in tear secretions were observed in doses of 5, 50 and 100 µg/ml capsaicin. Specifically, tear secretion following 5, 50 and 100 µg/ml capsaicin exposure were 4.22 ± 0.23, 3.12 ± 0.36 and 2.34 ± 0.66 mm/min versus 6.4 ± 0.45 and 6 ± 0.09 mm/min in control and vehicle treated rats respectively. Values have been depicted as mean ± standard deviation, n = 6.

Diminished tear fluid secretion might be due to decreased afferent input responsible for the reflex that increases such secretion in response to corneal injury^21^. Previous studies involving treatment of rat pups with capsaicin has shown decreased nociceptive stimuli and peripheral sensitivity as a result of decreased tear secretions^30^. Kagawa *et al*.^14^, have found that capsaicin induced reductions in tear secretions have induced superficial punctate keratopathy and led to a sustained decrease of corneal sensitivity^13^.

### Transcorneal permeation

The corneal permeation profile of 50 mg/ml capsaicin sample versus dexamethasone (standard) across bovine corneas is shown in Fig. 5(b). The steroid dexamethasone, widely used as an ocular topical anti-inflammatory agent, is highly lipophilic in nature and was used as a reference standard for comparative permeation studies of capsaicin^31^. Capsaicin showed rapid and instantaneous permeation across the corneal membrane initially until the 1^st^ hour, which was followed by a linear and gradual decrease in its permeability rate after the 2^nd^ and 3^rd^ hours. 4 hours after exposure, there was little significant permeation. The standard dexamethasone, on the other hand, showed a similar pattern of permeation with maximal permeation at the first hour followed by a gradual decrease later. The permeability rate exhibited rapid and fast absorption of about 53.8% capsaicin (26.9 mg/cm^2^) and 45.8% (22.9 mg/cm^2^) dexamethasone in the first hour. Accordingly, the steady state flux (Jss) was measured from the slope of the permeation curve as 0.3630 ± 0.214 mg/cm^2^ and the corresponding permeability coefficient (Kp) was found to be 0.4031 ± 0.309 mg/cm^2^ for capsaicin permeation. These values were higher in capsaicin treated corneas as compared to the dexamethasone permeated corneas, for which the Jss and Kp values were obtained as 0.2850 ± 0.211 and 0.3164 ± 0.242 respectively. Values have been depicted as mean ± standard deviation, n = 4.

Published literature have reported the passage of substances across the bodily membranes to be influenced by membrane properties like thickness, pores, effective surface area and the physico-chemical characteristics of the drug administered^32,33^. Previous studies have shown increased amounts of dexamethasone reaching different eye tissues when supplied as dexamethasone-cyclodextrine microparticles via the ocular topical route^31^. Though lipophilic, dexamethasone has shown limited permeability via the topical route and is known to show excellent absorption when administered as an intravitreal injection for retinal delivery^34^. The corneal epithelium, more specifically, is physiologically and partially impervious to polar compounds with relative molecular weight greater than 200–300Daltons^35^. Contrary to that, lipophilic compounds (of considerable molecular size) can pass the epithelium as they easily solubilise in the lipid cell membranes^36,37^. Capsaicin, being a lipophilic irritant compound with a fair permeability profile (including a consistent permeability coefficient and membrane flux properties) across corneal membranes, therefore, finds particular utility for defence purposes where it is considered more effective, faster acting, less toxic yet quite capable of provoking temporary incapacitation.

### Scanning Electron Microscopy (SEM) analysis of bovine corneas

**Figure 6:**
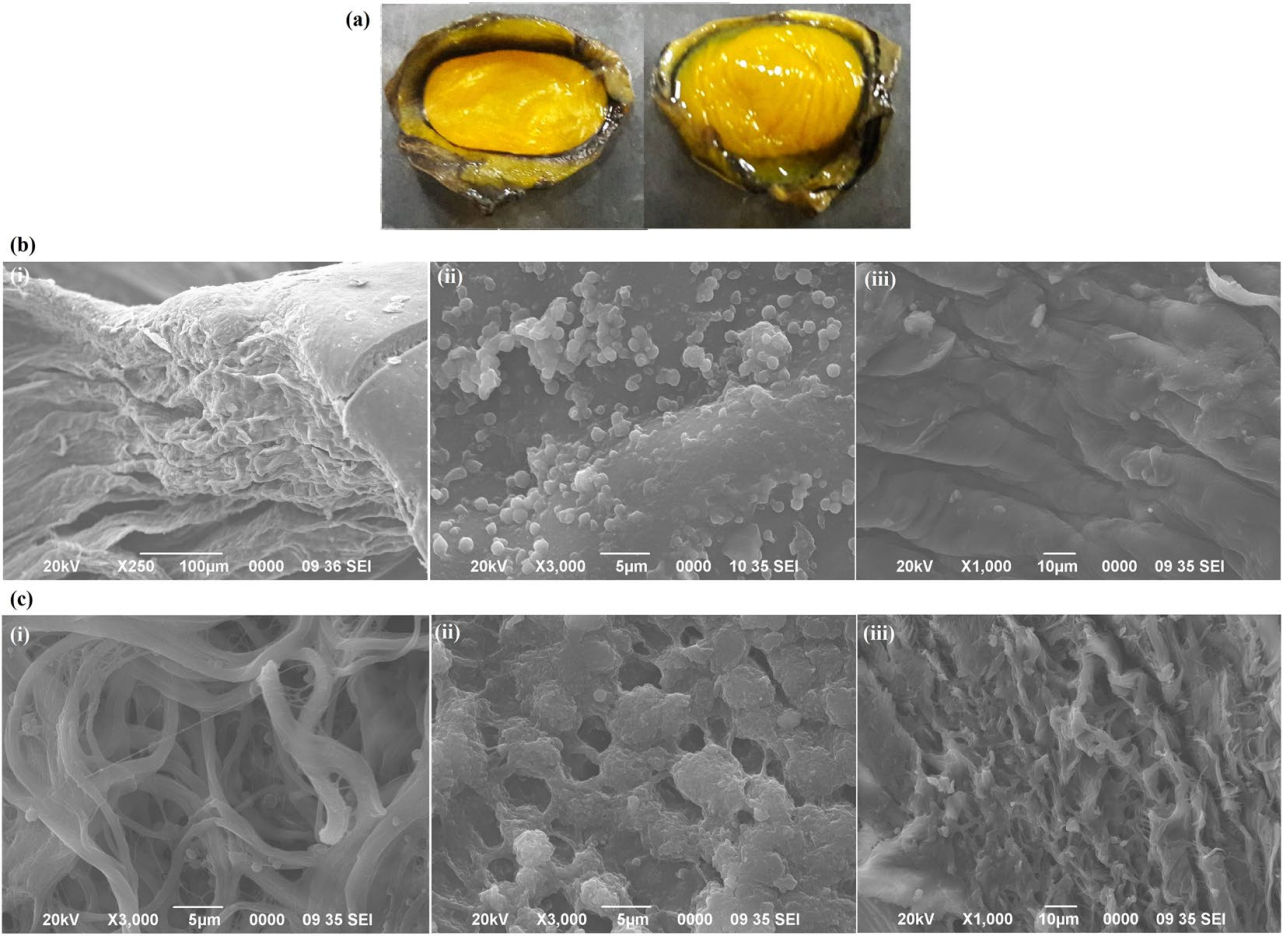
(**a**) Schematic image of bovine cornea: epithelial and endothelial sections (**b**) SEM images of control bovine corneas: (i) epithelial hole like structures with a raised border (x250 magnification) (ii) clusters of red blood cells on the corneal endothelial surface (x3000) (iii) regular and smooth stromal surfaces with muscular cushion like features (x1000). (**c**) SEM imaging of 50 mg/ml capsaicin treated bovine corneas: (i) structures resembling bundles of corneal sensory nerve fibres (x3000) (ii) raised areas of polygonal cells located centrally across the corneal surface (x3000) (iii) unsmooth and disorganized stromal surface containing micro folds and peripheral irregularities (x1000).

SEM of the 50 mg/ml capsaicin exposed bovine corneas [Fig. 6(a)] showed little morphological disfiguration when compared to the control corneas. In scattered locations, epithelial hole like structures with a raised border (x250 magnification) revealed several ridge like cellular arrangements on this layer [Fig. 6b(i)]. Figure 6b(ii) shows circular clusters of red blood cells on the corneal endothelial surface (x3000). Control corneas exhibited regular and smooth stromal surfaces (x1000) with muscular cushion like features as shown in Fig. 6b(iii). Structures resembling bundles of corneal sensory nerve fibres (x3000) were observed in capsaicin treated corneas [Fig. 6c(i)]. Treated corneal tissues (x3000) showed raised areas of polygonal cells [Fig. 6c(ii)] that were located centrally with a circular profile and formed a fairly level sheet across the corneal surface, which appeared similar when magnified and compared with to the polygonal cells obtained in Fig. 6b(ii). Treated stromal surface (x1000) was not smooth or regular and contained micro folds and peripheral irregularities as seen in Fig. 6c(iii).

As studied by Chandler *et al*.^38^, the ultrastructure of bovine corneal epithelium, being a stratified squamous variety, resembles other species such as human, porcine etc^38^. Existing studies on normal bovine corneas have helped us integrate, intensify and compare our results to some extent. Studies by Pfister, 1973 have revealed scattered locations on bovine corneas containing epithelial holes^39^, the description of which is quite similar to our obtained image [Fig. 4b(i)]. Also, such structural stromal surface exhibiting polygonal cells have been previously reported in cattle^40^. SEM images of our treated tissues bear resemblance to previous data obtained by Feher *et al*., 2009, who revealed the protective effects of Pigment epithelium-derived factor (PEDF) on retrobulbar treatment of capsaicin on rats’ eyes^41^.

### Electrophysiological study: nerve conduction velocity (NCV)

**Figure 7:**
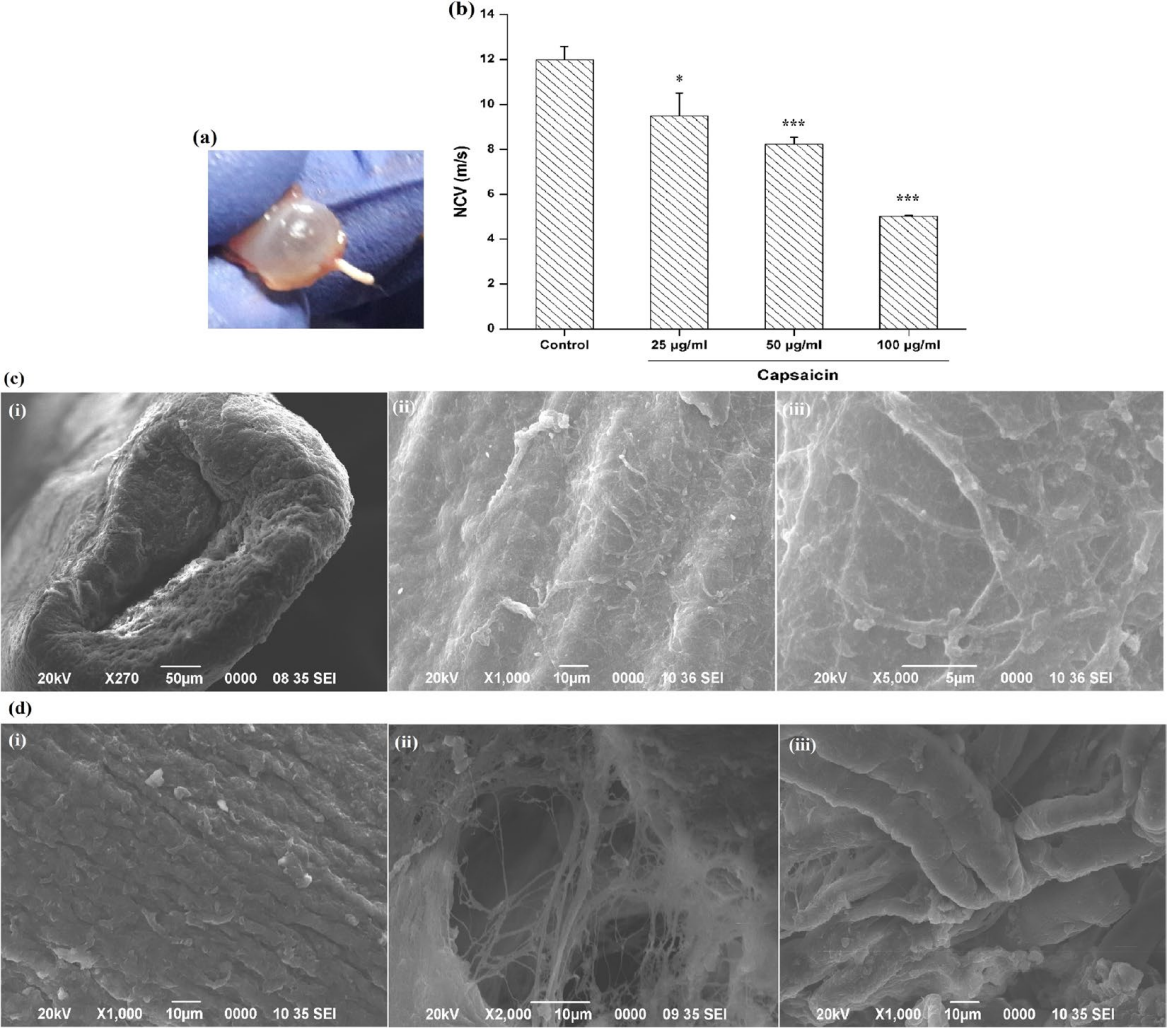
(**a**) Schematic image of a rat optic nerve (**b**) Nerve conduction velocity: graph indicates variations in conduction of nerve impulses across the control as well as 25, 50 and 100 µg/ml capsaicin treated rat optic nerves. Each vertical bar shows the mean ± standard deviation (n = 4). Statistically significant differences were observed in capsaicin treated groups when compared with the control groups (^***^P < 0.001, ^*^P < 0.05). (**c**) SEM imaging of control optic nerve: (i) longitudinal cross section of a normal optic nerve (x270 magnification) (ii) the same at a higher magnification (x1000) (iii) the control optic nerve head surface richly supplied with a dense network of blood vessels (x5000). (**d**) SEM imaging of 50 µg/ml capsaicin treated optic nerve: (i) Cytoplasmic extensions of optic nerve surface smooth morphological organization (x1000) (ii) possible disruption of spherically arranged neurofilaments of collagen fibers observed at the level of the optic nerve (x2000) (iii) meninges/cushion like structures which are the blood vessels surrounding the nerve (x1000).

The effect of 25, 50 and 100 µg/ml of capsaicin on the conduction of nerve impulses across rat optic nerves (Fig. 7a) was examined by supramaximally stimulating the retinal end of the nerves to record compound action potentials (CAP) and then calculating the NCV using the Biopac hardware and software systems (Biopac Systems, Inc, California, USA). Stimulator and response potentials curves were recorded from the conduction experiment. Action potential variations in particular sections of stimulator and corresponding response curves (for both control and treated nerves) were noted and the average conduction velocities for each nerve was calculated from it. The NCV of control nerve was 12 ± 0.128 m/s as compared to NCVs of 9.50 ± 0.891, 8.24 ± 0.564 and 5.02 ± 0.253 m/s for capsaicin treatments of 25, 50 and 100 µg/ml respectively (Fig. 7b). Values are mean ± standard deviation (*n* = 4). Differences of means were statistically significant (*P < *0.05) when compared with controls. Obtained results clearly indicate significant reduction of velocity in axonal nerve conduction in capsaicin treated optic nerves when compared to the velocities in control nerves. Thus, our experimental observation of variations in the conduction of a nerve impulse along the optic nerve when exposed to different concentrations of capsaicin resulted in reduced velocities in capsaicin treated nerves.

Earlier reports claim damage to the myelin sheath indicated by the slowing of NCV^15^. However, experimental analysis has not revealed any injury/damage to the myelin sheath. Exposure/treatment method as well as the time of irritant exposure are some factors that might influence the amount of damage caused to the nerve. There have also been no previous reports suggesting capsaicin induced demyelination on optic nerves. Previous electrophysiological examinations of nerve conduction in rat nerves exposed to capsaicin have yielded conflicting results^42^. Wall & Fitzgerald (1981) reported no effects of capsaicin on nerve conduction whilst Petsche *et al*. (1983) concluded substantial conduction block in afferent C-fibres^43,44^. Previous experiments reporting such decreased nerve conduction velocity include a study by Conradi *et al*.^45^, who observed reduced NCV in optic nerves following early post-natal low-dose lead exposure^45^.

### SEM analysis of optic nerve

Anatomical modifications in 50 µg/ml capsaicin exposed rat optic nerves was studied by SEM analysis. Figure 7c(i) is a longitudinal cross section of a normal optic nerve when observed at a low magnification (x270 magnification). Figure 7c(ii) and at a higher magnification, Fig. 7c(iii) shows the control optic nerve head surface (at x1000 and x5000 magnifications respectively) richly supplied with a dense network of blood vessels including large retinal vessels and venous tributaries. However, no choroidal artery/vein was observed. Cytoplasmic extensions (x1000) of capsaicin treated optic nerve surface can be seen in Fig. 7d(i), which appear regular and have quite smooth morphological organization. Treated nerve cross sections (x2000) indicate possible disruption of spherically arranged neurofilaments of collagen fibers observed at the level of the optic nerve [Fig. 7d(ii)]. Figure 7d(iii) shows the meninges/cushion like structures (x1000) which are nothing but the blood vessels surrounding the nerve that act as a direct extension from the eye to the brain.

Scanning electron microscopy data from control as well as treated optic nerves revealed little significant morphological changes in them. Slight disorganization and disruption of collagen fibre bundles was observed in the treated nerve which was somewhat familiar to what Joos *et al*., 2010, have described earlier^46^. They have mentioned intraocular pressure induced disruption of neurofilaments paired with disorganized myelin sheaths and enlargement of the axoplasmic space in rat optic nerve. Similar to our observed data, branches of retinal arteries sharply angulating away and small lobular nodes divided by right-angle branching within the retina were observed by Risco *et al*.^47^, and Hayreh, 2011^47,48^. May and Drecoll, in 2002, studied the morphology of the murine optic nerve where they confirmed the lack of lamina cribrosa and particularly, choroidal vascular supply, the latter of which was also observed from this study^49^.

### Enzyme-linked immunosorbant assay (ELISA)

**Figure 8:**
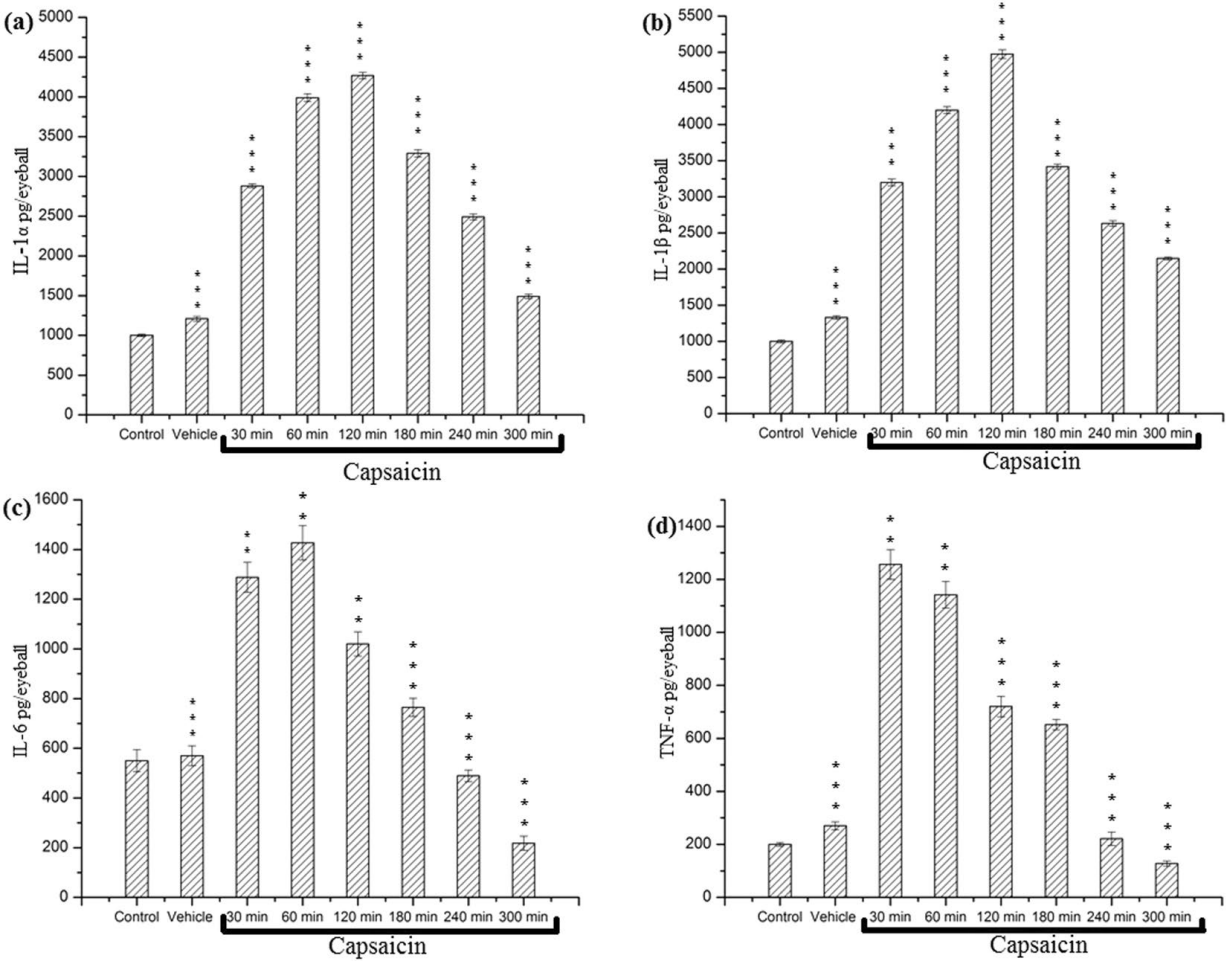
Enzyme-linked immunosorbant assay: (**a**) IL-1α (**b**) IL-1β, (**c**) IL-6 (**d**) TNF- α. Each bar in the graph represents the values of mean ± standard deviation, n = 4. Statistically significant differences were observed in capsaicin treated groups when compared with the control groups (^***^P < 0.001, ^**^P < 0.01). For each treatment, measurements were made at each time interval on a separate group of animals following ocular exposure to 50 µg/ml of capsaicin. Results showed significant upregulation of specific proinflammatory cytokine levels thereby indicating their aid in the inflammatory cascade.

Cytokine levels in ocular tissue homogenates of both control as well as 50 µg/ml of capsaicin exposed rats were determined by ELISA which showed significant upregulation of specific proinflammatory cytokine levels (Fig. 8). The levels of interleukins IL-1α and IL-1β showed significant increases that peaked at around 2 hrs and recovered to their basal levels at 4–5 h (Fig. 8a,b), whereas IL-6 peaked sooner at 1 hr and showed recovery after 4 h (Fig. 8c). The tumour necrosis factor-α, TNF- α peaked at 1/2 hr and showed recovery after 4 h (Fig. 8d). Compared to their levels in control animals, the level of IL-1α increased from 1045 ± 0.211 to 3990 ± 0.335 pg per eyeball, the IL-1β level increased from 1000 ± 0.131 to 4269 ± 0.423 pg per eyeball, the IL-6 level increased from 550 ± 0.822 to 1427 ± 0.127 pg per eyeball and the TNF-α level increased from 200 ± 0.348 to 1256 ± 0.250 pg per eyeball. Thus, capsaicin induced significant increments in the levels of assessed cytokines (P < 0.05). Values are presented as mean ± standard deviation, n = 4. All comparisons have been made with reference to values observed in control animals, which were close to those observed in animals exposed to the vehicle.

Previous studies by Biro *et al*.^50^ have shown that under special *in vitro* conditions, capsaicin can activate non-neuronal tissues^50^, which might facilitate exudation of associated proteins from actin microfilaments and microtubules present in the ocular cytoskeleton. Another explanation of the observed moderate increase of cytokine levels in the eyeball could be due to the massive discharge produced by the cutting of its optic nerve, similar to suggestions by Saade *et al*.^51^, who confirmed capsaicin induced neurogenic inflammation and upregulation of proinflammatory cytokines induced by bacterial toxins and other inflammatory agents^51–53^. Capsaicin is known to cause the local release of neuropeptides such as substance P, calcitonin gene-related peptide etc, which interfere with one or more cellular mechanisms involved in the inflammatory cascade and might be possibly be a cause increased cytokine secretion^54,55^. Varied inflammatory instances, based on experimental evidences such as aforementioned, might act as contributing factors to a better understanding of the phenomena behind pro-inflammatory cytokine secretion.

## Conclusion

In summary, the present research is an attempt at studying some elementary pharmacological effects of ocular capsaicin. The AEI test demonstrated precise details of capsaicin induced irritation expressions while the BCOP assay confirmed its impact on opacity and permeability in bovine corneas. A combined AEI and BCOP analysis was effective in reviewing the UN GHS categorization of capsaicin. Corneal fluorescein staining revealed no significant ocular surface damage by capsaicin whereas indirect ophthalmoscopy exhibited its  prominent effects on the inner eye influenced by its dosage. Transcorneal permeation study revealed considerable permeation of capsaicin across corneal membranes. Reductions in tear secretion and nerve impulse conduction across rat optic nerves, increments in proinflammatory cytokine levels are some features that confirm capsaicin’s active participation in the inflammatory cascade. Histopathological study and SEM analysis of bovine corneas and nerves served as supplementary aids as they contributed towards better prediction of the pharmacology of eye irritation induced by capsaicin. The overall pharmacology of ocular capsaicin, therefore, proves it as a safe, non-lethal and indispensable part of defence/military utility purposes as opposed to other predominantly available but hazardous products such as 2-chlorobenzalmalononitrile (CS) and 2-chloroacetophenone (CN) sprays.

## Materials and Methods

### Ethics statement for the use of experimental animals in research

Wistar rats (*Rattus norvegicus*) were used in all animal experiments. For AEI testing, New Zealand albino rabbits (*Oryctolagus cuniculus*) were used to study capsaicin induced ocular irritation. All experimental animals were used in accordance with ethical guidelines from the Defence Research Laboratory, Tezpur, Assam, India under the Institutional Animal Ethics Committee’s (IAEC) Approval number: 1227/bc/07/CPCSEA, approved on: 04/05/2016. The authors hereby affirm adherence to the Association for Research in Vision and Opthalmology (ARVO) statement for the use of animals in opthalmic and vision research. We have attempted to minimize the number as well as sufferings of animals during experimentation.

### Animals

All experiments employed albino Wistar rats (200–250 gms) as experimental animals. Animals of both the sexes were used during study. The temperature and relative humidity of the animal room was maintained at 20 °C (±3 °C) and 50–60% respectively. They were fed a conventional laboratory diet and provided with an unlimited supply of drinking water. New Zealand albino rabbits (2–3 kgs), used in AEI testing, were housed in same controlled environments as the rats and a 12 hr light and dark cycle was maintained. They were fed with standard laboratory feed and were provided free access to unlimited supply of drinking water. Animals that were healthy and free of any clinically observable ocular surface disease were used for the study.

### Materials

Capsaicin samples (CAS: 8023-77-6) were kindly gifted by Ozone Naturals, Haryana, India and was common for all the experiments discussed here. Dimethyl sulfoxide (DMSO) (Sigma Aldrich, CAS no: M81802) was used as vehicle to prepare the capsaicin dilutions. All the other experimental chemicals were of reagent grade and were used as received without further purification.

### Methods

#### Tiered approach for assessing eye irritation

Oliveira *et al*. had combined the Short Time Exposure (STE) test with BCOP assay to assess the correspondence of two particular textile dyes with their Globally Harmonized System (GHS) eye irritation classification^12^. The current determination of the irritation potential of capsaicin might be a novel technique as it accomplishes and associates the results of the AEI and BCOP tests as a tiered approach to evaluate the UN GHS categorization of capsaicin.

#### Acute eye irritation

Rabbits: The OECD TG 405 acute eye irritation/corrosion testing strategy was followed^10^. Young and healthy adult New Zealand Albino rabbits (*Oryctolagus cuniculus*) were used for experiment. Capsaicin being a serious ocular irritant, 3 male rabbits were used for the initial test followed by 3 additional animals for the confirmatory testing. Both eyes of the experimental animals selected for testing were examined for 24 hours before start of the test. The left eye of the rabbit was treated with 50 mg/ml capsaicin; while the right eye served as control. The dose of capsaicin was chosen in accordance with previously reported data by Vesaluoma *et al*.^56^. The lower lid of the eyeball was pulled gently and capsaicin solution was placed in its conjunctival sac for 5 seconds. The lids were held together to prevent the loss of the solution inside the sac. The eye of the animal was not washed for the next 24 hours following instillation of capsaicin. The eyes were evaluated for the existence of ocular lesions one hour post capsaicin applications followed by observations at specific intervals of 24 hrs, 48 hrs and 72 hrs respectively. Grading of lesions was done with the aid of personnel adequately trained in the ocular scoring system.

Rats: Wistar rats were used, in addition to rabbits, as a complementary study to evaluate the ocular irritation potential of capsaicin in varied doses in their eyes. Using rats for this experiment was more convenient considering the evaluation of responses based on different doses of capsaicin, which is not the case with rabbits. In rabbits, the use of varied doses means the use of more animals, which is not acceptable as per ethical guidelines. Rats were divided into four groups, each group consisting of six animals and exposed to capsaicin treatments of 25, 50, 75 and 100 µg/ml using similar procedure as for rabbits. Little acute lethality data of ocular capsaicin is available^57^, based on which we have chosen a considerable dose essential for ocular irritation experimentation. Rats were evaluated for the existence of ocular lesions one hr post capsaicin application followed by observations at specific intervals of 24 hrs, 48 hrs and 72 hrs respectively.

#### Histopathology

Eyes from control rats as well as 25, 50, 75 and 100 µg/ml of capsaicin treated groups were enucleated and preserved in10% formalin (Sigma, CAS- HT501128). Macroscopic corneal transverse cross sections were made and dehydrated in ethanol (70–100%), cleared using xylene (Sigma, 214736) and finally embedded in paraffin. A microtome (Spencers Semi Automatic Rotary Machine) was used to prepare sections of about 3 micrometers. The slides containing ocular sections were stained using Hematoxylin (Sigma, CAS No-HHS32) & Eosin (Sigma, CAS- HT110132) staining and observed under the light microscope at 40X to examine for any other signs of inflammatory response.

#### BCOP assay

The BCOP analysis was carried out as per the OECD TG 437 guidelines^13^. It was performed to examine the rate or extent of opacity and permeability in the corneal membranes when exposed to different doses of capsaicin, i.e, 5, 25 and 50 mg/ml. Briefly, eyes collected at a nearby slaughterhouse (shortly after death) were immersed in Hanks’ Balanced Salt Solution (HBSS) (Sigma, CAS- H9394) and preserved on ice during collection and transported to laboratory. Corneas were isolated, mounted in corneal holders and brimmed to an excess with lukewarm Eagle’s Minimum Essential Medium (EMEM) (Sigma, CAS- M2279) in the posterior chamber. For 1 hr, the device was readjusted at 32 ± 1°C and holders were calibrated at 74, 150 and 224 opacity units. The corneas were exposed to capsaicin dilutions prepared in dimethyl sulfoxide (DMSO). After 2 minutes of exposure, controls, PBS 7.4 (Sigma, CAS-P3813), Benzalkonium chloride 5% (Sigma, CAS- PHR1371) and test samples were removed from the anterior chamber and the epithelium was washed three times to eliminate any excess capsaicin that might have remained. This was done to avoid any erratic interference in the spectrophotometer measurement. An opacitometer (DURATEC Analysentechnik GmbH, Germany) was used to measure the corneal responses by decreased light transmission or corneal opacity. Transportation of sodium fluorescein dye (Sigma, CAS- 30181) across the control and capsaicin treated corneas was studied to evaluate their comparative permeability and integrity. 1 ml of sodium fluorescein (4 mg/mL) solution, prepared in PBS 7.4, was placed in the anterior compartment of the corneal holder and is adjusted such that the corneal holder interfaces with the corneal epithelia, while the posterior compartment interfaces with the corneal endothelial. Fresh EMEM was placed in the posterior compartment. The quantity of sodium fluorescein passing into the posterior compartment was measured quantitatively with a UV Visible Spectrophotometer (Shimadzu, UV-2600) at 490 nm. An *In Vitro* Irritancy Score (IVIS) is useful in allocating the irritancy level of a test group as mentioned previously in the results section. Further BCOP histopathological examination (similar processing of tissues as mentioned previously) was also carried out for the better characterization and complementation of the depth and severity of corneal irritation^13^.

#### Corneal fluorescein staining

2 μl of freshly prepared 1% fluorescein sodium (prepared in double distilled water) was applied topically into the conjunctival sac of control as well as DMSO exposed, 25, 50, 75 and100 mg/ml of capsaicin exposed groups of rabbits, n = 3. Ocular surface of the animals after fluorescein treatment were inspected using the cobalt blue filter of the slit-lamp microscope. The degree of fluorescein staining in the temporal and nasal conjunctiva and cornea was evaluated. A standardized 4-point scale (0 = none, 1 = mild, 2 = moderate, and 3 = severe) was applied in each of three areas to analyze the results^27^.

#### Indirect opthalmoscope imaging

Control as well as DMSO exposed, 25, 50, 75 and 100 mg/ml of capsaicin exposed rabbit eyes were observed using a Keeler Vantage Indirect Ophthalmoscope. Pupils of rabbits from each group (n = 3) were dilated with 1% tropicamide eye drops and rabbits were positioned in a convenient restraining equipment for observation. The Vantage opthalmoscope headband was put on and the attached eyepiece on the ophthalmoscope was vertically aligned to observe the rabbit eyes for signs of inner eye damage. A condensing lens (20 Dioptre) was fixed in the light path approximately 1 inch from the animal’s pupil and a red retinal reflex was obtained on directing the light beam at the pupil. Observed images of the fundus were captured using the Vantage Plus Digital software system provided with the instrument.

#### Schirmer tear secretion test

For the measurement of tear fluid secretion, the animals were divided into 5 groups: control, vehicle and 5, 50 and 100 µg/ml capsaicin exposed groups. Tear fluid secretion was measured without topical anesthesia by the Schirmer tear test method. Rats were exposed to topical capsaicin and tear fluid secretion both before and after capsaicin exposure was estimated. Schirmer tear strips (Alcon Laboratories, Item #4050) were inserted into the lower eyelid of the eye without topical anesthesia. 1 min later, the wetted length of the strip (millimetres) was observed and taken as the test score.

#### Transcorneal permeation

Bovine eyes obtained from the local slaughter house were suspended in HBSS. Fresh corneas were isolated from the eyes. 50 mg/ml dexamethasone was used as the standard drug to study permeation of 50 mg/ml of capsaicin sample in a Franz diffusion apparatus. Both samples were allowed to permeate across the corneal membrane placed between the donor and receptor compartment of the Franz diffusion cell with an effective diffusion area of 1.130 cm^2^ and PBS 7.4 (same as lacrimal fluid) as the media at 37 ± 2 °C. The amount of capsaicin and dexamethasone permeated across the membrane was assessed by the intermittent sampling of 5 ml of receptor media at predetermined time intervals and each time replaced with fresh reservoir fluid. The cumulative amount of drug permeated through the corneal membrane was quantified by a UV-visible spectrophotometer (Shimadzu UV-2600, Japan).

#### Permeation data analysis

Fick’s law of diffusion was used for the estimation of membrane flux. The expression Jss = dQt/Adt was used, where Jss denotes the steady-state flux in mg/cm^2^ per hour, dQt denotes changes in the amount of capsaicin passing through the corneal membrane into the receptor compartment in mg, A denotes the active diffusion area in cm^2^ and dt denotes the time variation. Permeation profiles were built by plotting the cumulative amount of drug permeated per unit area of the corneal membrane (mg/cm^2^) versus time (h). The steady state flux (Jss; mg/cm2/h) was obtained from the slope of the plot. The equation Kp = Jss/C, where C denotes the concentration of capsaicin present in the sample for permeation analysis, was used for the calculation of the permeability coefficient (Kp; cm^2^/h).

#### SEM analysis of cornea

The bovine corneas were subjected for SEM analysis to check for any variations in corneal morphology on acute exposure to capsaicin spray (50 mg/ml). Untreated corneas served as control for the SEM analysis. Primary fixation was done using 2.5% gluteraldehyde (Sigma, G5882) for 4 hr. The corneas were subjected to further secondary fixation using 1% Osmium tetroxide (Sigma, 75632) for 4 hrs for better penetration. Corneal cross sections were made by slicing at a length of 1 mm using glass cutter in a microtome maintaining uniformity. The sliced nerve segments were further observed under a scanning electron microscope (JEOL, Japan, model: JSM 6390LV) for any structural changes.

#### NCV study

NCV is commonly the velocity at which an electrical stimulus travels through the nerve fibres that constitute the nerve. For this experiment, rats were divided into 4 groups: control and 25, 50 and 100 µg/ml capsaicin exposed groups. The treated groups were exposed to capsaicin spray topically for 5 seconds. The optic nerves from rats were then extracted and analysed for NCV using the Biopac hardware and software setup systems. Fresh nerve was placed in a conduction chamber irrigated with artificial cerebrospinal fluid having a composition as follows: NaC1, 123.9 mM; KCI, 5.0 mM; MgSO4, 1.7 mM; CaCI, 1.9 mM; NaHCOs, 26.0 mM; D-glucose, 9.9 mM; saturated with 95% O_2_, 5% CO_2_ (pH −7.4)^58^. These chemicals were all purchased from Sigma Aldrich, USA. This chamber was attached with a low voltage stimulator (SS58L). To record the conduction of an impulse, the stimulation was increased at increments of 0.1 Volt (maximum upto 1 Volt) until a compound action potential (CAP) response was obtained. Nerve conduction in the normal optic nerves versus capsaicin treated nerves were recorded on a data acquisition BSLCBL4B system connected to transducers and amplifiers to record the nerve response. NCV generated along the length of the nerve was then calculated using the following formula:

$$NCV=\frac{{\rm{L}}{\rm{e}}{\rm{n}}{\rm{g}}{\rm{t}}{\rm{h}}\,{\rm{o}}{\rm{f}}\,{\rm{t}}{\rm{h}}{\rm{e}}\,{\rm{n}}{\rm{e}}{\rm{r}}{\rm{v}}{\rm{e}}\,({\rm{m}}{\rm{m}})}{{\rm{T}}{\rm{i}}{\rm{m}}{\rm{e}}\,{\rm{t}}{\rm{a}}{\rm{k}}{\rm{e}}{\rm{n}}\,{\rm{f}}{\rm{o}}{\rm{r}}\,{\rm{c}}{\rm{o}}{\rm{n}}{\rm{d}}{\rm{u}}{\rm{c}}{\rm{t}}{\rm{i}}{\rm{o}}{\rm{n}}\,({\rm{m}}{\rm{s}}{\rm{e}}{\rm{c}}{\rm{s}})}$$

#### SEM analysis of optic nerve

Optic nerves of rat exposed 50 µg/ml of capsaicin as well as controls were isolated and analysed for SEM analysis using procedure similiar to the SEM procedure of bovine corneas.

#### ELISA assay

Rats belonging to both control and 50 µg/ml capsaicin exposed groups were treated with an additional anaesthetic dose and killed by cervical dislocation. Whole rat eyeballs were removed and 5 mL of PBS 7.4 was added. Eyeballs were weighed accurately and homogenized in PBS 7.4 containing 0.4 M NaCl, 0.05% Tween 20, 0.5% bovine serum albumin, 0.1 mM phenylmethyl- sulphonyl-fluoride, 0.1 mM benzethonium chloride, 10 mM EDTA and 20 IU aprotinin^51^. The used chemicals were all purchased from Sigma Aldrich, USA. Centrifugation of homogenates at 12000 revolutions per minute (RPM) was done for 30 min at 4 °C. The levels of various cytokines such as IL-1α, IL-1β, IL-6 and TNF-α were measured by performing ELISA using the supernatant obtained from these homogenized tissues. Reagent kits for IL 1α, IL-1β, IL-6 and TNF- α were purchased from Sigma Aldrich, USA. Briefly, each cytokine estimation procedure followed was the same as for IL-1α, which has been mentioned below. All reagents were brought to room temperature (18–25 °C) before use. 100 µl each of standards (lyophilized rat IL-1α protein standard) and samples were added to the rat IL-1α antibody-coated microwells of a 96 well immunoplates (Sigma). Samples were added in triplicate. Covered wells were kept for overnight incubation at 4 °C with gentle shaking. The solution was later discarded and microwells washed with 1X wash solution 4 times. 100 µl of freshly prepared biotinylated rat IL-1α Detection antibody was then added to appropriate wells and incubated with gentle shaking for 1 hour at room temperature. The wells were discarded after 1 hour and washed as before. 100 µl of freshly prepared Horse radish peroxidise, i.e, HRP-Streptavidin solution was added to appropriate wells and incubated with gentle shaking for 45 minutes at room temperature. The wells were discarded, washed and 100 µl of colorimetric Tetramethylbenzidine (TMB) reagent was added to appropriate wells and incubated in the dark with gentle shaking for 30 minutes. 50 µl of stop solution was then added to appropriate wells and the microplate was read at 450 nm immediately using a microplate reader (Spinco Biotech Pvt. Ltd., India). The cytokine values obtained from each animal group at the indicated time intervals was averaged and variations in cytokine levels assessed.

#### Statistical analysis

Data obtained from AEI, BCOP, tear secretion, transcorneal permeation, NCV and ELISA experiments were expressed as mean ± standard deviation (S.D). Analysis of comparative means was done by one-way analysis of variance (ANOVA) by utilizing the Tukey Kramer multiple comparisons test (GraphPad InStat). All P values < 0.05 were considered statistically significant.

## Data Availability

All data generated or analysed during this study are included within this article.
